# On the Enlisting, Discharging and Pensioning of Soldiers, with the Official Documents

**Published:** 1839-10

**Authors:** 


ART. VIII.—*On the Enlisting, Discharging, and Pensioning of Soldiers, with the Official Documents on these branches of Military Duty.* By HENRY MARSHALL, F.R.S.E., Deputy Inspector-General of Army Hospitals. *Second Edition.—Edinburgh,* 1839. 8vo, pp. 259.

THIS is a most valuable book, and ought to be in the library of every medical officer in the public service, whether of the army or navy. "In a financial, a political, and, perhaps, I may add, in a medical point of view, I am not aware," says the author, "of any part of the duty of a medical officer which is of more importance than the inspection of recruits on a large scale and the examination of inefficient soldiers; and consequently these duties deserve a very careful consideration." We can add, that in Mr. Marshall's pages will be found the completest and most efficient aid for enabling the officer to discharge this duty with accuracy. Under the head of "Diseases and Disabilities, which disqualify Soldiers for service in the Army," is given a comprehensive and interesting view of the feigned and factitious diseases of the class of men termed *malingerers* in the army and *skulkers* in the navy, which we would recommend not merely to the notice of officers in the public service, but to the medical staff of our civil hospitals and workhouses.

